# Molecular and Functional Significance of Growth Differentiation Factor-15: A Review on Cardiovascular-Kidney-Metabolic Biomarker

**DOI:** 10.2174/011573403X332671241121063641

**Published:** 2025-01-07

**Authors:** Krishna Tiwari, Aswini Saravanan, Abhishek Anil, Vikas Kumar Tiwari, Muhammad Aaqib Shamim, Surjit Singh, Pradeep Dwivedi, Surender Deora, Shoban Babu Varthya

**Affiliations:** 1 Department of Pharmacology, All India Institute of Medical Sciences, Jodhpur, Rajasthan, 342005, India;; 2 Laboratory of Systems Neuroscience, Tohoku University Graduate School of Medicine, Sendai, Japan;; 3 Department of Physiology, JIET Medical College and Hospital, Jodhpur, India;; 4 Department of Cardiology, All India Institute of Medical Sciences, Jodhpur, Rajasthan, 342005, India

**Keywords:** GDF-15, diagnostic marker, prognostic marker, therapeutic target, cellular functions, molecular functions, cardiovascular-kidney-metabolic syndrome

## Abstract

Cardiovascular-kidney-metabolic (CKM) syndrome is the association between obesity, diabetes, CKD (chronic kidney disease), and cardiovascular disease. GDF-15 mainly acts through the GFRAL (Glial cell line-derived neurotrophic factor Family Receptor Alpha-Like) receptor. GDF-15 and GDFRAL complex act mainly through RET co-receptors, further activating Ras and phosphatidylinositol-3-kinase (PI3K)/Akt pathways through downstream signaling. GDF-15 decreases cardiac dysfunction and hypertrophy by inducing HIF-α (hypoxia-inducible factor-1α). It causes increased fractional shortening and a significant decrease in ventricular dilation through the induction of the SMAD 2/3. GDF-15 prevents hyperglycemia-induced apoptosis in diabetes mellitus. GDF-15 causes anorexia by influencing the central systems regulating metabolism and appetite. Therefore, targeting GDF-15 can be useful for the treatment of anorexia caused by cancer as well as the prevention of resulting weight loss. GDF-15 has an important role in predicting mortality in acute kidney injury. Its high levels are related to eGFR decline and also have a prognostic role in CKD patients. Growth differentiation factor-15 (GDF-15) is a vital biomarker for diagnosis, treatment, and prognosis of CKM syndrome. Elevated GDF-15 levels can be utilised as a biomarker to determine the suitable metformin dosage. In light chain amyloidosis, a raised level of GDF-15 predicts early death in heart failure and renal disease patients. *In vivo*, studies using GDF-15 analogs and antibodies against GFRAL to affect metabolic parameters and ventricular dilatation have shown potential for GDF-15-based therapeutic interventions. This review aims to study the role of GDF-15 in CKM syndrome and establish it as a CKM biomarker.

## 1 INTRODUCTION

Cardiovascular-kidney-metabolic (CKM) syndrome is a recently coined term that explains the connection between obesity, diabetes, chronic kidney disease (CKD), and cardiovascular disease (CVD), but a clear definition is yet to be explained in detail [1].

Cardiovascular diseases, renal disorders, and metabolic conditions are deeply interconnected through shared pathways of oxidative stress, inflammation, and mitochondrial dysfunction. Growth differentiation factor 15 (GDF-15) plays an important role in linking these disorders by acting as a stress-responsive cytokine with regulatory functions across these systems. This ability of GDF-15 to bridge cardiovascular, renal, and metabolic disorders makes it an essential focus for both diagnosis and prognosis in CKM, offering insights into disease severity and progression. This review focuses on the diagnostic, prognostic, and therapeutic role of GDF-15 as a biomarker in CKM syndrome.

GDF-15 is a cytokine, sometimes referred to as Macrophage inhibitory cytokine [2], NSAID-activated gene 1 protein (NAG-1) [3], Placental TGF-β [4], Placental bone morphogenetic protein [5], and Prostate differentiation factor [6]. GDF-15 was identified from a subtracted cDNA containing genes related to macrophage activation [7]. Valenzuela *et al*. reported that the expression of a clone isolated from the subtraction library was similar to transforming growth factor β (TGF-β) superfamily [8]. To check the similarity with the TGF- β superfamily, Bauskin *et al*., performed dna star program, which compared the pairwise sequence. The comparison showed that seven cysteine domains, also known as cystine knot of GDF-15, are highly conserved and have almost 30% similarity to TGF- superfamily members [9]. The human locus of GDF-15 is present at chromosome 19p12.1-13.1 when mapped by fluorescence *in situ* hybridization [4].

## 2 STRUCTURE

GDF-15 is released as a pre-pro-peptide containing 308 amino acids, where the signal sequence contains 29 amino acids, the pro-peptide includes 167 amino acids, and the mature peptide has 112 amino acids [10]. The 3D crystal structure of the GDF-15 prepropeptide is shown in Fig. (**1**), along with the labeled first amino acid positioning of three segments [11, 12].

## 3 SEQUENTIAL MATURATION OF GDF-15

Pre-pro-peptide is an inactive precursor of GDF-15, whose main role was thought to be for the accurate folding of mature peptides. Frameshift deletion of pro-peptide results in intracellular accumulation of disulfide-linked aggregated and inhibited secretion of mature peptide dimer in all TGF- members [13]. In contrast, when similar frameshift deletion was done in GDF-15, no such effect of accumulation of aggregates was seen. Then, this non-aggregation was supposed to be due to the absence of cysteine residue in the GDF-15 pro-peptide. Then, the importance of pro-peptide was later linked with the biological properties of mature peptide, where pro-peptide is non-covalently bound with mature peptide.

Further, inhibits the biological activity of the GDF-15 [9]. Later, the disulfide-linked dimerization of pre-pro peptides through a cysteine residue forms, proteolytic cleavage by protease, and matrix metalloproteinases occur for further maturation [14]. This cleaving happens in the Golgi body, usually at 193^rd^ or 196^th^ in the amino acid chain [15, 16]. The characteristic that makes GDF-15 unique is having two additional cysteines that are present in the carboxyl-terminal domain that lead to dimerization through disulfide linkage [17]. The stepwise maturation of GDF-15 is shown in Fig. (**2**).

## 4 TISSUE DISTRIBUTION OF GDF-15

GDF-15 is present in higher levels in the placental, prostate, kidney, and liver physiologically. However, there is low expression in other organs [18]. When physiological levels were assessed using immunoassays, the range was found to be 400–3000 ng/L, while in pregnancy, levels were around >19,000 pg/mL [19, 20]. Fig. (**3**) shows the RNA expression of GDF-15 in various organs [21, 22]. GDF-15 values are raised in pathological disorders such as cancer, liver diseases, kidney diseases, metabolic diseases, and cardiovascular disorders [23, 24]. To obtain levels of GDF-15, serum samples were stored at -80°C and measured by enzyme‐linked immunosorbent assay (ELISA) [25].

## 5 REGULATION OF GDF-15

### 5.1 Transcription

At the GDF-15 promotor site, there are several areas where transcription factors such as specificity protein 1(Sp1), early growth response protein 1 (Egr- 1), p53, and COUP Transcription Factor 1 (COUP-TF1) bind during stress situations which further causes the release of GDF-15 [26-28]. Patel *et al*. explained that stress situation that imbalance leads to raised GDF-15 expression, which is regulated by transcriptional regulators like Activating Transcription Factor 4 (ATF4) and C/EBP homologous protein (CHOP) [3]. Hypoxia may lead to GDF-15 transcription by the eukaryotic initiation factor 2 alpha signaling pathway [29, 30]. Baek *et al*. studied NSAIDS leading to NAG-1 protein expression, which further induces GDF-15 *via* Egr- 1 transcription, whereas indomethacin induces GDF-15 in less amount [27]. The role of angiotensin II receptor antagonist in cardio-protection is mainly by inhibition of apoptosis, by downregulating p53, and lowering inflammation is mainly due to raised GDF-15 [31].

### 5.2 Downstream Signal Regulation

The major receptor for GDF-15 is Glial Cell Line-Derived Neurotrophic Factor Family Receptor Alpha-Like (GFRAL). GFRAL is exclusively localized in the hindbrain [17]. mRNA of GFRAL in the brainstem is seen in the area postrema and near nucleus tractus solitarius (NTS).

When GFRAL bounds to the intracellularly expressed domain of GDF-15, it has a binding constant of 3330 pM and binds with the extracellular domain with a constant value of 8 nM. This shows high affinity and direct interaction of GFRAL with the extracellular domain of GDF-15 [17, 32]. To check the GDF15–GFRAL interaction, the D1, D2, and D3 domains of GFRAL were eliminated individually. Protein expression is lowered when the D1 or D2 domain is inhibited. However, GDF-15 was coimmunoprecipitated with the D1 domain but not with the D2 domain, suggesting the D2 domain of GFRAL is important for its attachment to GDF-15 [33]. After GDF-15-GFRAL interaction, downstream intracellular signaling is further executed by the interaction of the GFRAL complex and rearranged during transfection (RET) tyrosine kinase co-receptor [34]. RET receptor autophosphorylation is performed by ligand binding, which further promotes differentiation of cells and survival by various downstream pathways, including Ras/ mitogen-activated protein kinase (MAPK) and phosphatidylinositol-3-kinase (PI3K)/Akt pathways [35]. Inactivated Ras is bound to Guanosine Diphosphate (GDP) and binds to Guanosine Triphosphate (GTP) when activated extracellularly. The extra phosphate in GTP holds the two switch regions. After the release of phosphate from GTP, it leads to an inactivated state by relaxing the switch region [36-38]. Upon Ras binding to GTP, it subsequently associates with Rapidly Accelerated Fibrosarcoma (Raf). It facilitates the translocation of the inactive protein from the cytoplasm to the plasma membrane through the involvement of Raf kinases [37]. Ras further activates Mitogen-Activated Protein Kinase 1 and 2 after transportation. This further activates effector Extracellular Signal-Regulated Kinase 1 and 2 (ERK1 and ERK2), which play a role in cell survival, differentiation, and motility by causing gene phosphorylation. Besides ERK1/2, Ras can activate PI3K and Rho proteins which regulate the cytoskeleton and invasiveness of tumor cells [38, 39]. Dual mechanisms promote cell survival. One is a posttranslational modification, which causes the inactivation of pathways responsible for cell death, and the other method is by the increasing transcription of pro-survival genes [40]. Another receptor for GDF-15 other than GFRAL is CCN2, which has a role in the increase in angiogenesis. This angiogenesis action of CCN2 is after the activation of Alpha V Beta 3 (a_V_b_3)_ integrins [41, 42]. Activation of a_V_b_3_ integrins plays a role in focal adhesions. The mediation of this action is facilitated by GDF-15, which was verified through the treatment of HUVEC with media containing CCN2 alone or in conjunction with GDF-15. The utilization of an antibody against the aVb3 integrin in immunofluorescence assays concluded an increase in punctate formation following treatment with CCN2 alone. However, this pattern was not observed when CCN2 was co-administered with GDF-15. This implies that GDF-15 may attenuate the formation of aVb3 integrin complexes, contrasting with the observed increase induced by CCN2 alone [43].

## 6 ROLE IN CARDIOVASCULAR DISEASES

### 6.1 Coronary Artery Disease

Cardiac myocytes release GDF-15 in response to oxidative, ischemic, and mechanical stress [44]. Cardiac dysfunction and dysregulation cause deterioration of mitochondrial performance and lead to increased generation of reactive oxygen species (ROS) [45]. The hypoxia leads to the upstreaming of the hypoxia-inducible factor -1α (HIF-1α) transcription factor. The exposure of cells to elevated oxygen levels restricts the functionality of HIF-1α. GDF-15 activates HIF-1α signaling through different pathways. GDF-15 activates HIF-1α signaling *via* different pathways. One method stabilizes the p53/ mouse double minute 2 (MDM2) complex, whereas another involves activating Akt, MAPK, and NF-kB signaling pathways [31, 46, 47]. This allows for the expression of three key regulators of mitochondrial dynamics: the optic atrophy type-1 GTPase (OPA-1), mitofusins family of GTPases (Mfns), and dynamin-related protein 1 dynamin-like GTPase (DRP1). The OPA-1 mutation causes a reduction of fractional shortening, a fall in cardiac output volume, and causes myocyte contraction. However, overexpressing OPA-1 results in physiological heart hypertrophy [48]. Mfns promote mitochondrial outer membrane fusion [49]. DRP1 leads to mitochondrial fission. This finding supports that mitochondrial fission contributes to the development of cardiovascular disorders by inhibiting GDF-15, which can be a potential target for cardio-protection. Mitochondrial fission contributes to the development of cardiovascular disorders by inhibiting GDF-15, which suggests that GDF-15 may have a cardio-protection role [50].

In Myocardial Infarction (MI), GDF-15 pro-peptide levels increased, reached maximum levels after 6 hours, and returned toward baseline after 12 hours. Mature peptide levels are also raised after reperfusion or ischemia [51]. After an episode of MI, the anti-inflammatory role of GDF-15 on the heart is through activating cell division control protein 42 (Cdc42) homolog, which later suppresses chemokine-triggered β2 integrin. Studies have indicated higher myeloid cell infiltration and cardiac rupture rates in mice with the GDF-15 knockout model. In the infarcted myocardium, the Matrix Metalloproteinase-9 (Mmp) levels are elevated by raised expression of GDF-15 [52]. Inhibition of Mmp-9 reduced the risk of ventricular rupture during MI [53]. Elevated levels of GDF-15 have been found to correlate with an increased risk of recurrent MI. Raised concentrations of GDF-15 in uncomplicated MI individuals suggest a worse prognosis over a year [54]. The invasive procedure, such as Percutaneous intervention, reduces around 50% risks of the composite endpoint composite of death or MI at 6 months in patients with GDF-15 levels ≥1200 ng/L. In contrast, patients with <1200 ng/L did not benefit much from invasive procedures [55]. Widera *et al*. predicted the prognosis of non-ST elevation Acute Coronary Syndrome (ACS) among various biomarkers. GDF-15 was superior to other biomarkers in adding prognostic information to the Global Registry of Acute Coronary Events score and hs-cTnT in patients with non-ST-segment elevation acute coronary syndrome [56]. These prognostic functions of GDF-15 have been demonstrated to be independent of other cardiac biomarkers like Brain Natriuretic Peptide (BNP). In a research experiment, an increase in GDF-15 expression was observed in the ischemic area when mice underwent coronary artery ligation. The mRNA levels escalated in under an hour, and this heightened state was maintained in the ischemic region for nearly a week. When coronary blood flow was restored after one hour of ischemia, it led to a gradual rise in both GDF-15 mRNA and pro-peptide in the ischemic region [51]. A meta-analysis revealed the correlation between GDF-15 levels and instances of hospitalization due to heart failure (HHF), cardiovascular death, and mortality from all causes. However, the relationship between GDF-15 and ischemic events was not as pronounced [57]. GDF-15 suppresses lipid accumulation in oxidized LDL-treated macrophages. Additionally, GDF-15 decreased the inflammatory response in oxidized LDL-treated macrophages. These findings collectively indicate that GDF-15 may play a role in inhibiting the initiation and progression of atherosclerosis [58].

### 6.2 Heart Failure

GDF-15 and BNP levels predict increased mortality risk in heart failure (HF) cases, irrespective of whether the ejection fraction (EF) is preserved or reduced. This may be due to the shared pathology of cardiac hypertrophy and fibrosis between all types of heart failure. It was further demonstrated that in HF patients with preserved EF and mildly reduced EF, GDF-15 was independent and superior to BNP in assessing the prognosis [59, 60]. In the DAN-MONICA trial involving 3785 patients, a comparison was made between C reactive protein, Cystatin C, and GDF-15 as prognostic biomarkers. This was done by determining their C-indices for heart failure models. They concluded GDF-15 played a significant role in evaluating mortality from heart disease, and C reactive protein was identified as a predictor for heart failure [61]. Xu *et al*. conducted an *in vivo* experiment done in mice, and explained that GDF-15 has a role in altering heart failure and ventricular dilation. The conclusion was drawn that introducing recombinant GDF-15 enhanced fractional shortening and a notable reduction in ventricular dilation. This was achieved through the significant activation of the SMAD 2/3 phosphorylation pathway [62]. SMAD 2/3 are subjected to phosphorylation, following which they transition into the cell nucleus. SMAD2 and SMAD3 can engage with one another, and this interaction is dependent on TGF-β [63]. SMAD 4 forms complexes with SMAD2 and SMAD3, and full transcriptional activity is achieved when all come together as a complex. Overexpression of SMAD 2 inhibits cardiac hypertrophy, whose mechanism was similar to hypertrophy caused by exogenous GDF-15. However, over-expression of the inhibitory SMAD proteins, SMAD6/7, antagonizes the antihypertrophic effects of GDF-15 [62]. Xu *et al*. determined that the molecular foundation of GDF-15 interactions, which are crucial for GDF-15 activity, is associated with GFRAL. This, in turn, triggers the activation of RET, as opposed to TGFβ signaling [17]. Rochette *et al*. explained that signaling mediated by TGF-β through the SMAD pathway in the adult heart regulates cellular growth, proliferation, and inflammation [31]. However, more evidence is needed to determine the pathway of GDF-15 activity in the heart. GDF-15 has a role in erythropoiesis as it causes hepcidin downregulation, leading to iron overload and ineffective erythropoiesis [63, 64]. Garimella *et al*. explained that raised erythropoietin levels were associated with an increased risk of HF. Doubling of erythropoietin levels was significantly associated with almost 30% higher risk of HF [65]. GDF-15 and BNP levels are predictive indicators for evaluating the increased mortality risk in heart failure cases, irrespective of whether the ejection fraction is preserved or reduced. This may be due to the shared pathology of cardiac hypertrophy and fibrosis between reduced and preserved heart failure. It was further demonstrated that in heart failure (HF) patients with preserved ejection fraction (EF) and mildly reduced EF, GDF-15 was independent and superior to BNP in assessing the prognosis [59, 60]. In the DAN-MONICA trial involving 3785 patients, a comparison was made between C reactive protein, Cystatin C, and GDF-15 as prognostic biomarkers. This was done by determining their C-indices for heart failure models. The conclusion drawn was that GDF-15 played a significant role in evaluating mortality from heart disease, and C reactive protein was identified as a predictor for heart failure [61]. Xu *et al*. conducted *in vivo* experiment on mice and explained that GDF-15 has a role in altering heart failure and ventricular dilation. The conclusion was drawn that introducing recombinant GDF-15 enhanced fractional shortening and a notable reduction in ventricular dilation. This was achieved through the significant activation of the SMAD 2/3 phosphorylation pathway [62]. SMAD 2 and 3 are subjected to phosphorylation, following which they transition into the cell nucleus. SMAD 4 forms complexes with SMAD 2 and SMAD 3, and full transcriptional activity is achieved when all come together as a complex. SMAD 2 and SMAD 3 can engage with one another, and this interaction is dependent on TGF-β [63]. Overexpression of SMAD 2 inhibits cardiac hypertrophy, whose mechanism was similar to hypertrophy caused by exogenous GDF-15. However, over-expression of the inhibitory SMAD proteins, SMAD6/7, antagonizes the antihypertrophic effects of GDF-15 [62]. Xu *et al*. determined that the molecular foundation of GDF-15 interactions, which are crucial for GDF-15 activity, is associated with GFRAL. This, in turn, triggers the activation of RET, as opposed to TGFβ signaling [17]. Rochette *et al*. explained that signaling mediated by TGF-β through the Smad pathway in the adult heart regulates cellular growth, proliferation, and inflammation [31]. However, more evidence is needed to determine the pathway of GDF-15 activity in the heart. GDF-15 has a role in erythropoiesis as it causes hepcidin downregulation, leading to iron overload and ineffective erythropoiesis [64]. Garimella *et al*. explained that raised erythropoietin levels were associated with an increased risk of HF. Doubling of erythropoietin levels was significantly associated with almost 30% higher risk of HF [65].

### 6.3 Light Chain (AL) Amyloidosis

Elevated GDF-15 showed a significant and independent role in prognosis for overall mortality over other biomarkers such as NT-proBNP and TnT, where elevated biomarkers indicate a worse prognosis in AL-amyloidosis [66]. A randomized controlled trial with 309 AL amyloidosis patients concluded that GDF-15 level >7575 pg/mL predicted early mortality [44]. GDF‐15 levels differed in patients with the different transthyretin genotypes of amyloidosis. Plasma GDF‐15 levels in patients with late‐onset transthyretin amyloidosis were higher than in patients with early‐onset amyloidosis [67].

## 7 RENAL DISORDERS

GDF-15 is expressed in the nephrons and collecting ducts [68]. When prognostic parameters were compared for predicting hospital mortality in acute kidney injury patients, GDF-15 was the most sensitive and specific when compared independently by integrating with other parameters [69]. Raised GDF-15 levels in CKD are significantly associated with a decline in the estimated glomerular filtration rate (eGFR) [70]. Patients in the high serum GDF-15 had more severe kidney disease and lower eGFR. However, patients with high urine GDF-15 had no significant differences in eGFR, but rather had lower hemoglobin [71]. In research conducted across two separate cohorts, it was found that elevated levels of GDF-15 were linked to more than a two-fold increase in the risk of eGFR decline in patients with chronic kidney disease (CKD). Elevated levels of GDF-15 in CKD can be correlated with intra-renal tubulointerstitial abnormality [70]. In a study, circulating levels of GDF-15 were measured in AL amyloidosis patients before and at 3 and 6 months after treatment initiation. They found that patients having more or equal to 4000 pg/ml have rapid progression to dialysis compared to patients with serum GDF-15 levels less than 4000 pg/ml [44].

## 8 ROLE IN METABOLISM

### 8.1 Diabetes Mellitus

CKM is linked to two main metabolic disorders: Type 2 Diabetes Mellitus (T2DM) and obesity, both of which are becoming increasingly prevalent [72]. The effect of hyperglycemia in T2DM can be explained in 2 phases. The initial phase is the inducer phase, characterized by raised glucose levels, further leading to a rise in Diacylglycerol synthesis. Phase II also called the effector phase, leads to the generation of Advanced Glycation End Products (AGEs) through oxidases. The complex of AGEs and the Receptor for AGEs can trigger both afferent and efferent signaling pathways. Both pathways subsequently activate NADPH-oxidase, increasing ROS production and decreasing Nitric Oxide (NO) levels, MAPK activation, and NF-kB activation [73]. GDF-15 protects against cellular apoptosis triggered by high glucose levels. GDF-15 inhibits the activation of the NF-kB/JNK pathway, thereby stimulating the PI3K/AKT/eNOS signaling pathway. This mechanism helps to maintain cellular health in high-glucose conditions. GDF-15 plays this role in a negative feedback manner [74]. Apelin, an adipose tissue-derived signal molecule, is associated with energy metabolism. Higuchi *et al*. found that treatment with apelin led to an enhancement of insulin sensitivity. When the group treated with saline was compared to the apelin group, they noticed a significant reduction in serum insulin, free fatty acids, and triglyceride levels. However, there was no evident reduction in body weight [75]. The role of GDF-15 in type 1 diabetes mellitus (T1DM) is explained by Nayakasu *et al*., who observed raised levels of GDF-15 in islets of individuals with T1DM individuals. Recombinant GDF-15 treatment reduced the incidence of T1DM by almost 53% in diabetic animal models [76]. While immune-mediated β-cell dysfunction is the primary pathology in T1D, and metabolic stress is the main pathology in Type 2 Diabetes Mellitus (T2DM), endoplasmic reticulum stress is a common pathology shared by both T1DM and T2DM. This highlights the complex interplay of various factors in the onset and progression of diabetes [77]. Al‐kuraishy *et al*. explained the role of GDF-15 in decreasing hyperglycemia‐induced oxidative stress and inflammation by nuclear factor kappa B (NF‐κB) [78]. The HOMA-IR study established that reduced insulin resistance corresponded with increased apelin levels. Furthermore, it was found that metformin treatment led to a decrease in insulin resistance. This highlights the potential role of apelin and metformin in managing insulin resistance. They explained that drugs that enhance insulin sensitivity could trigger the release of apelin by activating AMPK (adenosine 5′ monophosphate–activated protein kinase [79]. Insulin resistance is associated with decreased M2 macrophages, and GDF-15 mediates increased oxidative function in macrophages [80, 81]. So, GDF-15 also indirectly decreases insulin resistance. In the Outcome Reduction with Initial Glargine Intervention (ORIGIN) trial, a comprehensive analysis of 237 biomarkers was conducted on the baseline serum samples from 8,401 participants, including 2,317 individuals undergoing metformin treatment. This trial showed an association between the use of metformin and GDF-15 concentration [82]. Mazagova *et al*. investigated the role of GDF-15 in both T1DM and T2DM. Gene deletion of GDF-15 increases renal tubular and interstitial damage in both T1DM and T2DM models, and increased interstitial and tubular damage further causes glucosuria and polyuria. This concludes that GDF-15 and its reno-protective role are protective in T1DM and T2DM [83].

### 8.2 Anorexia

Johnen *et al*. demonstrated that the onset of anorexia is triggered by GDF-15 through its interaction with the Transforming Growth Factor β Receptor Type 2 (TGFβRII). This interaction subsequently leads to the phosphorylation of ERK 1/2 and the activation of the Transducer and Activator of Transcription 3 (STAT3) within the hypothalamus [84]. The activation of STAT3 leads to a dual effect: it inhibits the function of Neuropeptide Y neurons while simultaneously stimulating Pro-opiomelanocortin neurons. The interaction of GDF-15 with GFRAL triggers the activation of its co-receptor RET. This activation sequence results in the phosphorylation of ERK and Phospholipase C γ. The culmination of these biochemical events is a reduction in appetite, a common symptom in individuals suffering from cachexia [85]. So, by influencing the hypothalamus transforming growth factor-receptor II and signal transducer, GDF-15 impacts the central systems that regulate metabolism and appetite [84]. Therefore, GDF-15 presents itself as a potential therapeutic target for treating anorexia associated with cancer. It could also be used to prevent weight loss by enhancing the activity of the GFRAL-RET pathway (Glial Cell-Derived Neurotrophic Factor Family Receptor Alpha-Like-Ret Proto-Oncogene), which plays a significant role in lipid metabolism [85]. Moreover, it also escalates energy expenditure through GFRAL and beta-receptors. GDF-15 synergizes with leptin in reducing fat content and body weight [86]. Targeting GDF-15 plays a crucial part in anorexic patients by preventing weight loss caused by cancers [87]. In Higuchi *et al*., the change in expression of adiponectin mRNA when compared in the apelin group to the saline group was insignificant. The increasing change in serum adiponectin level was significant in the apelin group [75]. Suriben *et al*. performed calorimetry to assess the respiratory exchange ratio in calorie-restricted tumor-bearing mice. Their findings revealed a significant rise in lipid oxidation and a decline in glucose oxidation during the tumor-induced weight-loss process. These results further validate that lipolysis is the driving force behind cancer-induced GDF-15 cachexia and underscore the potential effectiveness of antibodies targeting GFRAL in reversing this weight loss [85]. Mice with a GFRAL knockout gene exhibit resistance to the appetite-reducing effects of exogenously administered GDF-15, which establishes the GDF15-GFRAL axis as critical to stress pathway-induced weight loss [17]. Minamino *et al*. suggested that the relation between obesity, aging, and irregular metabolism could be attributed to p53. Their research revealed that the absence of p53 reduced inflammation and enhanced insulin sensitivity in obese mice [88]. GDF-15 is a direct transcriptional target of p53. Therefore, p53 is an important link between obesity and insulin resistance [89]. Therefore, detecting a link between GDF-15 and insulin resistance might indicate a state of metabolic disorder.

## 9 AS THERAPEUTIC TARGET IN CKM

Various *in vivo* studies have created long-acting agonists, fusion proteins, and mutant glycol-variants of GDF-15. Few studies have also observed the effect of GFRAL antibodies on body weight, triglycerides, food intake, cholesterol levels, caloric intake, and glucose tolerance. *In vivo* studies that observed the effect of GDF-15 are explained in Table **1**, although studies explaining the role of GDF-15 in cardiovascular disorders are limited [90-115].

## CONCLUSION AND FUTURE DIRECTION

CKM syndrome deals with many comorbidities together, and there are various biomarkers that play a role in determining the prognosis as well as diagnosis of CKM. GDF-15 has a role in various physiological and pathological processes, encompassing its involvement in metabolic disorders, cardiac dysfunction, and renal disorders. Its significance in CKM lies in its ability to regulate key processes across these systems, such as mitochondrial mechanisms, oxidative stress, and inflammation. In cardiovascular disease, GDF-15 is activated in response to ischemic damage and helps by regulating inflammation and preventing mitochondrial dysfunction. Similarly, in renal disorders, GDF-15 reduces tubular damage by modulating oxidative stress and inflammatory pathways. These shared mechanisms between the heart and kidneys make GDF-15 a central biomarker in CKM syndrome. Studies that have explored the diagnostic prognostic role of GDF-15 in CKM syndrome have been mentioned in Tables **2** and **3**.Other than its diagnostic and prognostic role, GDF-15 holds significant potential for therapeutic applications in CKM syndrome. Its role in metabolic regulation-by improving insulin sensitivity, regulating lipid metabolism, and controlling energy expenditure-suggests it could be used to manage conditions like diabetes and obesity, which are major contributors to CKM. Ongoing research into GDF-15-based therapies, such as long-acting agonists and fusion proteins can be utilised as new treatments targeting the underlying mechanisms of CKM syndrome.

## LIMITATIONS

There are many studies on the various diagnostic and prognostic aspects of GDF-15. Studies focusing on the therapeutic role of GDF-15 are very limited. The lack of large trials studying GDF-15 in various cardiovascular and metabolic disorders limits the generalizability of the findings of this article. This article is not a systematic review, so some articles may not be included. Although significant evidence shows the importance of GDF-15 in metabolism and appetite regulation, very few *in vivo* studies have focussed on the role of GDF-15 in cardiovascular and renal disorders. More evidence and experiments are needed for the research to explore the role of GDF-15.

## Figures and Tables

**Fig. (1):**
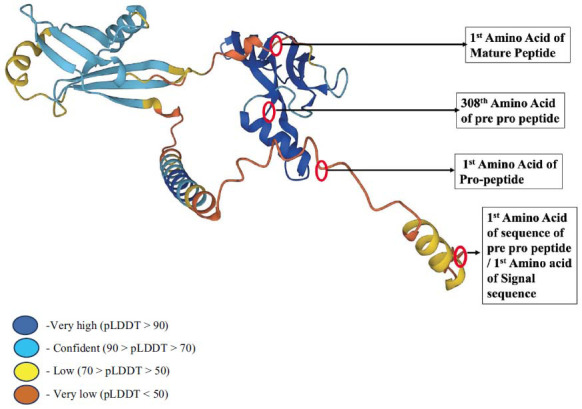
3D structure of pre-pro peptide of GDF-15 where various colour signifies model confidence. Amino acid marked with red circle points the place of specific amino acid of amino acid mentioned in 3d structure. Reprinted from AlphaFold Protein Structure Database, with permission from EMBL-EBI 2022.

**Fig. (2):**
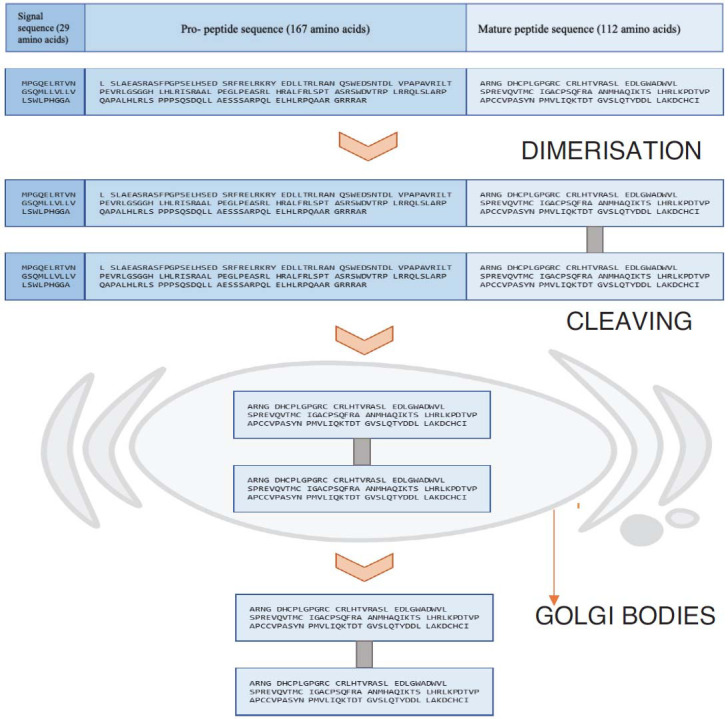
Depicts the sequential maturation of pre-pro peptide into mature peptide where various color shows different peptide chain containing amino acid sequence.

**Fig. (3):**
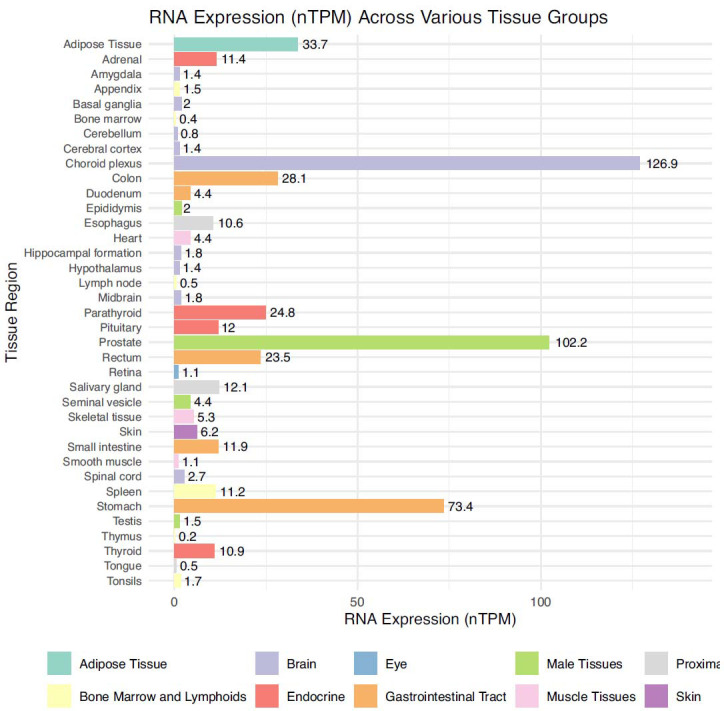
Figure shows the tissue RNA expression summary of GDF-15 in, where values are expressed in nTPM (transcripts per million) value represents the number of transcripts detected for a given tissue.

**Table 1:** Table depicting the studies explaining diagnostic role of GDF-15 in CKM.

Study	What the Study Aimed to Assess	Results
Kempf *et al*., 2009 [95]	How can GDF-15, either alone or as part of a multimarker strategy, improve risk prediction algorithms and support therapeutic management in patients with cardiovascular disease?	Elevated levels of GDF-15 can identify high-risk individuals across various cardiovascular conditions.
Lind *et al*., 2009 [96]	What is the relationship between GDF-15 levels and cardiovascular dysfunction and disease in the elderly population?	Elevated levels of GDF-15 were associated with various cardiovascular risk factors in the elderly. Increased levels of GDF-15 were linked to reduced endothelium-dependent vasodilation, plaque burden, left ventricular hypertrophy, reduced ejection fraction, and clinical signs of heart disease.
Gonzalez *et al*., 2020 [97]	What is the diagnostic potential of growth differentiation factor 15 in identifying mitochondrial diseases compared to other biomarkers?	GDF-15 is reliable diagnostic biomarker for mitochondrial diseases, showing high accuracy in diagnosis and correlation with disease severity and muscle biopsy findings.
Hagstrom *et al*., 2016 [98]	What is the association between GDF-15 levels and major bleeding, coronary lesions, and cardiovascular events in patients with acute coronary syndrome?	Higher levels of GDF-15 are associated with an increased risk of major bleeding, cardiovascular death, spontaneous myocardial infarction, stroke, and all-cause mortality in patients with acute coronary syndrome.
Eddy *et al*., 2021 [80]	What are the potential functions of GDF-15 and the signaling pathways implicated in its role in regulating metabolism, insulin sensitivity, and the cardiovascular system?	GDF-15 could be used as a biomarker for cardiovascular disease and diabetes, while also playing a protective role in regulating various functions and being cardioprotective in myocardial infarction.
Liu *et al*., 2013 [99]	What is the current knowledge and role of growth‐differentiation factor 15 (GDF‐15) in heart disease?	GDF-15 demonstrates properties of anti-hypertrophy of cardiomyocytes and protection of the heart from ischemia and reperfusion insult. cardiovascular diseases.
Wollert *et al*., 2012 [100]	What is the potential utility of GDF-15 in patient monitoring and treatment decisions in heart failure, and what new treatment targets in heart failure could be discovered through a better understanding of the pathobiology of GDF-15?	GDF-15 is associated with an increased risk of developing heart failure in apparently healthy individuals, elevated levels indicate a higher risk of adverse left ventricular remodeling and heart failure, and the information provided by GDF-15 is independent of established risk factors and cardiac biomarkers.
Montoro-Garcia *et al*., 2012 [101]	What is the relationship between GDF-15 levels and disease severity/functional status in patients with hypertrophic cardiomyopathy (HCM)?	Higher levels of GDF-15 are associated with conditions of severe disease in hypertrophic cardiomyopathy (HCM), suggesting GDF-15 as a novel marker related to disease severity and a potentially useful tool in monitoring functional capacity of HCM patients.
Kahli *et al*., 2014 [102]	What is the role of GDF-15 in the occurrence of organ dysfunction during coronary artery bypass grafting (CABG) associated with cardiopulmonary bypass (CPB)?	Plasma GDF-15 levels increased significantly during and after surgery, reaching nearly three times the IND levels in the ICU.
Eggers *et al*., 2012 [103]	What is the relationship between GDF-15 and biomarkers reflecting vascular pathologies in elderly subjects?	GDF-15 exhibited significant relations with biomarkers of endothelial activation in elderly subjects, indicating its role in reflecting endothelial activation and vascular inflammation.
Abdellah *et al*., [104]	What is the pattern of GDF15 gene expression in ACS patients with or without T2DM, and can GDF15 be a predictive biomarker for T2DM in ACS patients?	GDF15 expression is significantly higher in acute coronary syndrome patients compared to controls, with high sensitivity and specificity in predicting acute coronary syndrome. GDF15 could serve as a reliable sensitive biomarker for the prediction of acute coronary syndrome.
He *et al*., 2020 [105]	What is the correlation between serum GDF15 level and cardiovascular risk in patients with type 2 diabetes mellitus using the Framingham risk score?	Serum GDF 15 levels were significantly elevated in diabetic and prediabetic groups compared to controls, positively correlated with Framingham. Risk score, suggesting its potential as a clinical biomarker for cardiovascular risk in type 2 diabetes.
Carlsson *et al*., 2019 [106]	What are the associations between circulating proteins and prevalent DKD and major adverse cardiovascular events in individuals with type 2 diabetes?	Four proteins (KIM-1, GDF-15, myoglobin, and MMP-10) were positively associated with DKD in individuals with type 2 diabetes. Among these proteins, GDF-15 was significantly associated with an increased risk of major adverse cardiovascular events (MACE) in patients with DKD, even after adjustments for baseline factors, but this association was attenuated when further adjusted for cardiovascular risk factors.
Møller *et al*., 2018 [107]	Can GDF-15 and FGF-23 predict outcomes in patients with type 2 diabetes and microalbuminuria?	Evaluation of GDF-15 and FGF-23 as determinants of decline in eGFR, incident cardiovascular disease, and all-cause mortality in patients with type 2 diabetes and microalbuminuria, along with the provision of hazard ratios per 1 SD increment of log2-transformed values.
Na *et al*., 2017 [108]	Does Growth differentiation factor 15 (GDF 15) serve as a predictor of adverse renal outcomes in patients with immunoglobulin A nephropathy?	Growth differentiation factor 15 (GDF 15) has been identified as a useful prognostic marker in patients with chronic inflammatory disease and heart disease.
Kastritis *et al*., 2018 [44]	What is the prognostic importance of serum level of GDF-15 in patients with AL amyloidosis and how does it compare to traditional cardiac and renal risk biomarkers in predicting outcomes?	GDF-15 has prognostic implications for various outcomes in AL amyloidosis, including early death, overall survival, and progression to dialysis, independent of traditional cardiac and renal biomarkers.
Nair *et al*., 2017[70]	What is the association between circulating GDF-15 levels and decline in kidney function, as well as the correlation between circulating GDF-15 levels and intrarenal expression of GDF15?	Circulating GDF-15 levels are strongly correlated with intrarenal expression of GDF15 and associated with an increased risk of CKD progression. Circulating GDF-15 may be a marker for intrarenal GDF15-related signaling pathways in CKD.
Ham *et al*., 2018 [109]	How is growth differentiation factor-15 (GDF-15) associated with clinical parameters and disease progression in patients with idiopathic membranous nephropathy (IMN)?	Elevated GDF-15 levels were significantly associated with a higher risk of renal disease progression in patients with idiopathic membranous nephropathy (IMN), showing a negative correlation with initial renal function and providing useful prognostic information.
Thorsteinsdottir *et al*., 2020 [110]	What is the association between GDF-15 levels and cardiovascular risk factors in children with chronic kidney disease and after renal transplantation?	Plasma levels of GDF-15 are elevated in children with CKD and after renal transplant, strongly associated with renal function, but not useful as a biomarker for CVD in this population.
Perez Gomez *et al.,* 2021 [71]	What is the significance of urinary GDF15 levels as a biomarker of adverse outcomes and biopsy findings in chronic kidney disease?	Urinary GDF15 is associated with kidney histology patterns, mortality, and the need for renal replacement therapy in CKD patients who underwent a kidney biopsy.

**Table 2:** Table depicting the studies explaining prognostic role of GDF-15 in CKM.

Study	What the Study Aimed to Assess	Results
Wang *et al*., 2023 [111]	Can GDF-15 levels predict cardiovascular events and all-cause mortality in patients with stable coronary artery disease, independently of traditional risk factors and other biomarkers?	GDF-15 levels are predictive of cardiovascular events and all-cause mortality in patients with stable coronary artery disease. GDF-15 provides prognostic information independent of other biomarkers. GDF-15 could enhance risk prediction models for clinical events in secondary prevention for patients with stable CAD.
Kopystya *et al*., 2016 [112]	What is the association between serum GDF-15 levels and the diagnosis, risk stratification, and prognosis of cardiovascular diseases?	Patients with ACS and T2DM had a higher prevalence of cardiovascular diseases and risk factors, with significantly elevated GDF 15 levels in those with a history of T2DM.
Bao *et al*., [113]	What is the role of serum GDF-15 levels in the diagnosis, risk stratification, and prognosis of cardiovascular diseases?	The association between GDF-15 and diabetes risk was more pronounced in individuals without impaired fasting glucose and in those who were ≤60 years old at baseline.
Shin *et al*., 2016 [114]	What is the relation of GDF-15 to cardiovascular outcomes and how can it be used as a biomarker in cardiovascular disease?	Serum GDF 15 levels were significantly elevated in diabetic and prediabetic groups compared to controls, positively correlated with Framingham Risk Score, suggesting its potential as a clinical biomarker for cardiovascular risk in type 2 diabetes.
Kempf *et al*., 2012 [115]	What is the association between growth differentiation factor-15 and cardiovascular diseases?	Baseline GDF-15 levels were predictive of future prediabetes or diabetes and insulin resistance in obese nondiabetic individuals.
Na *et al*., 2017 [108]	Does Growth differentiation factor 15 (GDF-15) serve as a predictor of adverse renal outcomes in patients with immunoglobulin A nephropathy?	GDF -15 has been identified as a useful prognostic marker in patients with chronic inflammatory disease and heart disease.

**Table 3:** List of *in-vivo* studies showing the therapeutic role of GDF-15 on metabolic and cardiovascular disorders.

Study	Methodology	Result
Benichou *et al*. [90]	Subcutaneous administration of long-acting GDF-15 receptor agonist LY3463251 every 3 days for 2 weeks in normal Sprague Dawley (SD) rat.	Significant, dose-dependent loss of body weight and a prolonged decrease in food intake.
Benichou *et al*. [90]	A single high dose of LY3463251 in spontaneously obese cynomolgus monkeys.	Progressive decrease in body weight and food intake over 6 weeks. This weight loss was primarily due to a decrease in fat mass.
Xiong *et al*. [91]	Administered adeno- associated virus–expressing human GDF-15 in several obese mouse models.	Reduced food intake and body weight. The weight loss observed was primarily related to fat mass.
Xiong *et al*. [91]	In Dietary induced obese mice, daily treatment with recombinant human GDF-15 for 4 weeks	Decreased food intake, body weight, blood glucose, serum insulin, serum triglyceride, and cholesterol concentrations and improved glucose tolerance in a dose-dependent manner.
Xiong *et al*. [91]	Daily treatment with rhGDF15 for 6 weeks in obese cynomolgus monkeys.	Reduced food intake, body weight, plasma insulin, and plasma triglyceride concentrations and improved glucose tolerance
Xiong *et al*. [91]	Weekly dosing of fusion protein with a modified Fc deleted hinge region and fusion protein with one GDF-15 subunit per Fc dimer by incorporating a set of complementary charges in the CH3 domains of the Fc region in diet induced obese mice and cynomolgus monkeys.	Both fusion proteins reduced body weight, blood glucose, insulin, triglyceride levels, and food consumption in obese cynomolgus monkeys as reflected by a decrease in the glucose area under the curve during the oral glucose intake test was performed.
Fung *et al*. [92]	Fc-glyco variants of GDF-15: MUTANT 2C (N-glycan Mutant 2 (A3N/N5T) combined with up-mutation C (V98I) and l combination of N-glycan Mutant 3 (R4N/G6T)) And MUTANT 3B (N-glycan Mutant 3 (R4N/G6T) with up-mutation B (L36R)) in mice.	Mutant 2C (V98I) and Mutant 3B leads to improvements in production profile, protease resistance, and functional efficacy in but 2C was found to be more efficacious for weight loss.
Zhang *et al*. [94]	Effect of metformin infusion into the USI, ileum, or circulation on food intake, weight gain, and GDF15 levels and observing one hour after infusion.	1. Upper Large Vein (USI) Infusion: - Metformin reduced food intake and weight gain in the upper small intestine (USI) when rats were refed after a period of fasting compared to saline - The levels of GDF-15, a protein associated with appetite regulation, increased after infusion of USI metformin. - Metformin increased GDF-15 expression in the USI, ileum, and liver but not in the kidney. 2. Ileum graft: - Infusion of metformin into the ileum did not significantly affect food intake or weight gain compared with saline. Plasma GDF15 levels were not significantly increased after ileal metformin infusion. 3.Intravenous: - Metformin administered by intravenous injection resulted in decreased food intake and increased weight gain upon refeeding in mice. - Plasma GDF-15 levels increased after Intravenous metformin administration.
Suriben *et al*. [85]	Changes in food intake observed in mice implanted with HT1080 tumor bearing mice and compared with recombinant human GDF-15 mice.	Comparison to the reduction of food intake by recombinant GDF15, HT1080-tumor-bearing mice have only marginal reductions in cumulative food intake.
Suriben *et al*. [85]	when 3P10 (GFRAL antibody) was administered to HT1080-tumor-bearing mice	Mice gained back almost the entire amount of lost body and tissue weight, with only minimal changes in food intake.
Lim *et al*. [93]	Single subcutaneous injection of YH34160, which is an engineered GDF-15 variant-Fc fusion protein to have extended half-life and potent functional activity by enhancing binding affinity to GDF15 receptors (GFRAL/RET) in diet-induced obese mice.	YH34160-treated groups showed sustained and dose-dependent body weight reduction compared to long-acting glucagon like peptide-1 receptor agonist. Significantly better anti-obesity effects, and a much more improved metabolic profile. Combination with GLP-1RA or dual GLP-1/glucose-dependent insulinotropic polypeptide receptor agonist achieved more potent and greater BW loss compared to mono-therapy.
Xu *et al*. [62]	Tail vein infusion of Adβgal or AdGDF-15 in mlp null mice used a dosage of 5×108 plaque-forming units (11 days later mice were analyzed).	Adβgal-injected mlp−/− mice maintained depressed fractional shortening and dilation of the left ventricle, whereas AdGDF15-injected mlp−/− mice showed an increase in fractional shortening, as well as a significant decrease in ventricular dilation.
Xu *et al*. [62]	Twice daily injections of recombinant GDF-15 protein (10 μg each) for 14 days, compared with BSA (Bovine Serum Albumin) injections.	Partially reversed heart failure in mlp−/− mice, whereas control BSA injections had no significant effect.

## LIST OF ABBREVIATIONS

AAV-hGDF15: Adeno-associated Virus-expressing Human GDF-15
ACS: Acute Coronary Syndrome
AGEs: Advanced Glycation End Products
AKT: Protein Kinase B
ALK: Activin Receptor-like Kinase
ATF4: Activating Transcription Factor 4
BNP: Brain Natriuretic Peptide
BSA: Bovine Serum Albumin
Cdc42: Cell Division Control Protein 42
CHOP: C/EBP Homologous Protein
CKD: Chronic Kidney Disease
CKM: Cardiovascular-kidney-metabolic
COUP-TF1: COUP Transcription Factor 1
CRP: C-reactive protein
DNA: Deoxyribonucleic Acid
DRP1: Dynamin-related Protein 1
EF: Ejection Fraction
eGFR: Estimated Glomerular Filtration Rate
Egr-1: Early Growth Response Protein 1
GDF-15: Growth Differentiation Factor 15
GDP: Guanosine Diphosphate
GFRAL: Glial Cell Line-derived Neurotrophic Factor Family Receptor Alpha-like
GTP: Guanosine Triphosphate
HF: Heart Failure
HIF-α: Hypoxia-inducible Factor-1α
HUVEC: Human Umbilical Vein Endothelial Cells
IL-6: Interleukin-6
JAK/STAT1/3: Janus Kinase/Signal Transducer and Activator of Transcription 1/3
MDM2: Mouse Double Minute 2 Homolog
Mfn2: Mitofusins 2
MI: Myocardial Infarction
MMP: Matrix Metalloproteinase
NAG-1: NSAID-activated Gene 1
NF-kB: Nuclear Factor Kappa B
NT-proBNP: N-terminal Prohormone of Brain Natriuretic Peptide
NTS: Nucleus Tractus Solitarius
OPA-1: Optic Atrophy Type-1
PI3K: Phosphoinositide 3-Kinase
PKC: Protein Kinase C
Raf: Rapidly Accelerated Fibrosarcoma
RET: Rearranged During Transfection
ROS: Reactive Oxygen Species
Src: Proto-oncogene Tyrosine-protein Kinase
TGFβ: Transforming Growth Factor Beta
TnT: Troponin T

## References

[1] Ndumele C.E., Rangaswami J., Chow S.L. (2023). Cardiovascular-Kidney-metabolic health: A presidential advisory from the american heart association.. Circulation.

[2] Baek S.J., Kim K.S., Nixon J.B., Wilson L.C., Eling T.E. (2001). Cyclooxygenase inhibitors regulate the expression of a TGF-beta superfamily member that has proapoptotic and antitumorigenic activities.. Mol. Pharmacol..

[3] Baek S.J., Horowitz J.M., Eling T.E. (2001). Molecular cloning and characterization of human nonsteroidal anti-inflammatory drug-activated gene promoter. Basal transcription is mediated by Sp1 and Sp3.. J. Biol. Chem..

[4] Lawton L.N., Bonaldo M.F., Jelenc P.C. (1997). Identification of a novel member of the TGF-beta superfamily highly expressed in human placenta.. Gene.

[5] Hromas R., Hufford M., Sutton J., Xu D., Li Y., Lu L. (1997). PLAB, a novel placental bone morphogenetic protein.. Biochim. Biophys. Acta Gene Struct. Expr..

[6] Paralkar V.M., Vail A.L., Grasser W.A. (1998). Cloning and characterization of a novel member of the transforming growth factor-beta/bone morphogenetic protein family.. J. Biol. Chem..

[7] Harris P., Ralph P. (1985). Human leukemic models of myelomonocytic development: A review of the HL-60 and U937 cell lines.. J. Leukoc. Biol..

[8] Valenzuela S.M., Martin D.K., Por S.B. (1997). Molecular cloning and expression of a chloride ion channel of cell nuclei.. J. Biol. Chem..

[9] Bauskin A.R., Zhang H.P., Fairlie W.D. (2000). The propeptide of macrophage inhibitory cytokine (MIC-1), a TGF-β superfamily member, acts as a quality control determinant for correctly folded MIC-1.. EMBO J..

[10] Rochette L., Zeller M., Cottin Y., Vergely C. (2020). Insights into mechanisms of GDF15 and receptor GFRAL: Therapeutic targets.. Trends Endocrinol. Metab..

[11] Varadi M., Anyango S., Deshpande M. (2022). AlphaFold protein structure database: Massively expanding the structural coverage of protein-sequence space with high-accuracy models.. Nucleic Acids Res..

[12] Jumper J., Evans R., Pritzel A. (2021). Highly accurate protein structure prediction with AlphaFold.. Nature.

[13] Gray A.M., Mason A.J. (1990). Requirement for activin A and transforming growth factor - Beta 1 pro-regions in homodimer assembly.. Science.

[14] Dubois C.M., Laprise M.H., Blanchette F., Gentry L.E., Leduc R. (1995). Processing of transforming growth factor β 1 precursor by human furin convertase.. J. Biol. Chem..

[15] Wang X., Baek S.J., Eling T.E. (2013). The diverse roles of nonsteroidal anti-inflammatory drug activated gene (NAG-1/GDF15) in cancer.. Biochem. Pharmacol..

[16] Baek S.J., Eling T. (2019). Growth differentiation factor 15 (GDF15): A survival protein with therapeutic potential in metabolic diseases.. Pharmacol. Ther..

[17] Hsu J.Y., Crawley S., Chen M. (2017). Non-homeostatic body weight regulation through a brainstem-restricted receptor for GDF15.. Nature.

[18] Liu S., Chen X., Wang H. (2019). Association of GDF‐15 and syntax score in patient with acute myocardial infarction.. Cardiovasc. Ther..

[19] Wollert K.C., Kempf T., Giannitsis E. (2017). An automated assay for growth differentiation factor 15.. J. Appl. Lab. Med..

[20] Welsh P., Kimenai D.M., Marioni R.E. (2022). Reference ranges for GDF-15, and risk factors associated with GDF-15, in a large general population cohort.. Clin. Chem. Lab. Med..

[21] Uhlén M., Fagerberg L., Hallström B.M. (2015). Tissue-based map of the human proteome.. Science.

[22] https://www.proteinatlas.org/ENSG00000130513-GDF15/tissue.

[23] Emmerson P.J., Duffin K.L., Chintharlapalli S., Wu X. (2018). GDF15 and growth control.. Front Physiol.

[24] Koo B.K., Um S.H., Seo D.S. (2018). Growth differentiation factor 15 predicts advanced fibrosis in biopsy-proven non-alcoholic fatty liver disease.. Liver Int..

[25] Yatsuga S., Fujita Y., Ishii A. (2015). Growth differentiation factor 15 as a useful biomarker for mitochondrial disorders.. Ann. Neurol..

[26] Kim K.H., Kim S.H., Han D.H., Jo Y.S., Lee Y., Lee M.S. (2018). Growth differentiation factor 15 ameliorates nonalcoholic steatohepatitis and related metabolic disorders in mice.. Sci. Rep..

[27] Baek S.J., Kim J.S., Moore S.M., Lee S.H., Martinez J., Eling T.E. (2005). Cyclooxygenase inhibitors induce the expression of the tumor suppressor gene EGR-1, which results in the up-regulation of NAG-1, an antitumorigenic protein.. Mol. Pharmacol..

[28] Han M., Dai D., Yousafzai N.A. (2017). CXXC4 activates apoptosis through up-regulating GDF15 in gastric cancer.. Oncotarget.

[29] Patel S., Alvarez-Guaita A., Melvin A. (2019). GDF15 provides an endocrine signal of nutritional stress in mice and humans.. Cell Metab..

[30] Zheng H., Wu Y., Guo T. (2020). Hypoxia induces growth differentiation factor 15 to promote the metastasis of colorectal cancer *via* PERK-eIF2 α signaling.. Biomed Res. Int..

[31] Rochette L., Dogon G., Zeller M., Cottin Y., Vergely C. (2021). GDF15 and cardiac cells: Current concepts and new insights.. Int. J. Mol. Sci..

[32] Airaksinen M.S., Holm L., Hätinen T. (2006). Evolution of the GDNF family ligands and receptors.. Brain Behav. Evol..

[33] Emmerson P.J., Wang F., Du Y. (2017). The metabolic effects of GDF15 are mediated by the orphan receptor GFRAL.. Nat. Med..

[34] Yang L., Chang C.C., Sun Z. (2017). GFRAL is the receptor for GDF15 and is required for the anti-obesity effects of the ligand.. Nat. Med..

[35] Ibáñez C.F. (2013). Structure and physiology of the RET receptor tyrosine kinase.. Cold Spring Harb. Perspect. Biol..

[36] Díez D., Sánchez-Jiménez F., Ranea J.A.G. (2011). Evolutionary expansion of the Ras switch regulatory module in eukaryotes.. Nucleic Acids Res..

[37] Chong H., Vikis H.G., Guan K.L. (2003). Mechanisms of regulating the Raf kinase family.. Cell. Signal..

[38] Smeal T., Binetruy B., Mercola D.A., Birrer M., Karin M. (1991). Oncogenic and transcriptional cooperation with Ha-Ras requires phosphorylation of c-Jun on serines 63 and 73.. Nature.

[39] Binétruy B., Smeal T., Karin M. (1991). Ha-Ras augments c-Jun activity and stimulates phosphorylation of its activation domain.. Nature.

[40] Kolch W. (2005). Coordinating ERK/MAPK signalling through scaffolds and inhibitors.. Nat. Rev. Mol. Cell Biol..

[41] Lin C.G., Leu S.J., Chen N. (2003). CCN3 (NOV) is a novel angiogenic regulator of the CCN protein family.. J. Biol. Chem..

[42] Chen N., Leu S.J., Todorović V., Lam S.C.T., Lau L.F. (2004). Identification of a novel integrin alphavbeta3 binding site in CCN1 (CYR61) critical for pro-angiogenic activities in vascular endothelial cells.. J. Biol. Chem..

[43] Whitson R.J., Lucia M.S., Lambert J.R. (2013). Growth differentiation factor‐15 (GDF‐15) suppresses *in vitro* angiogenesis through a novel interaction with connective tissue growth factor (CCN2).. J. Cell. Biochem..

[44] Kastritis E., Papassotiriou I., Merlini G. (2018). Growth differentiation factor-15 is a new biomarker for survival and renal outcomes in light chain amyloidosis.. Blood.

[45] Sánchez-Díaz M., Nicolás-Ávila J.Á., Cordero M.D., Hidalgo A. (2020). Mitochondrial adaptations in the growing heart.. Trends Endocrinol. Metab..

[46] Song H., Yin D., Liu Z. (2012). GDF-15 promotes angiogenesis through modulating p53/HIF-1α signaling pathway in hypoxic human umbilical vein endothelial cells.. Mol. Biol. Rep..

[47] Dong G., Zheng Q.D., Ma M. (2018). Angiogenesis enhanced by treatment damage to hepatocellular carcinoma through the release of GDF 15.. Cancer Med..

[48] Varanita T., Soriano M.E., Romanello V. (2015). The OPA1-dependent mitochondrial cristae remodeling pathway controls atrophic, apoptotic, and ischemic tissue damage.. Cell Metab..

[49] Nan J., Zhu W., Rahman M.S. (2017). Molecular regulation of mitochondrial dynamics in cardiac disease.. Biochim. Biophys. Acta Mol. Cell Res..

[50] Ong S.B., Subrayan S., Lim S.Y., Yellon D.M., Davidson S.M., Hausenloy D.J. (2010). Inhibiting mitochondrial fission protects the heart against ischemia/reperfusion injury.. Circulation.

[51] Kempf T., Eden M., Strelau J. (2006). The transforming growth factor-β superfamily member growth-differentiation factor-15 protects the heart from ischemia/reperfusion injury.. Circ. Res..

[52] Kempf T., Zarbock A., Widera C. (2011). GDF-15 is an inhibitor of leukocyte integrin activation required for survival after myocardial infarction in mice.. Nat. Med..

[53] Heymans S., Luttun A., Nuyens D. Inhibition of plasminogen activators or matrix metalloproteinases prevents cardiac rupture but impairs therapeutic angiogenesis and causes cardiac failure.. Nat. Med..

[54] Galyavich A.S., Sabirzyanova A.A., Baleeva L.V., Galeeva Z.M. (2023). The role of growth differentiation factor-15 in assessing the prognosis of patients after uncomplicated myocardial infarction.. Kardiologiia.

[55] Wollert K.C., Kempf T., Lagerqvist B. (2007). Growth differentiation factor 15 for risk stratification and selection of an invasive treatment strategy in non–ST-elevation acute coronary syndrome.. Circulation.

[56] Widera C., Pencina M.J., Bobadilla M. (2013). Incremental prognostic value of biomarkers beyond the GRACE (Global Registry of Acute Coronary Events) score and high-sensitivity cardiac troponin T in non-ST-elevation acute coronary syndrome.. Clin. Chem..

[57] Kato E.T., Morrow D.A., Guo J. (2023). Growth differentiation factor 15 and cardiovascular risk: individual patient meta-analysis.. Eur. Heart J..

[58] Huang H., Chen Z., Li Y. (2021). GDF-15 suppresses atherosclerosis by inhibiting oxLDL-induced lipid accumulation and inflammation in macrophages.. Evid. Based Complement. Alternat. Med..

[59] Otaki Y., Shimizu M., Watanabe T. (2023). Growth differentiation factor 15 and clinical outcomes in Japanese patients with heart failure.. Circ. J..

[60] Mendez Fernandez A.B., Ferrero-Gregori A., Garcia-Osuna A. (2020). Growth differentiation factor 15 as mortality predictor in heart failure patients with non‐reduced ejection fraction.. ESC Heart Fail..

[61] Fluschnik N., Ojeda F., Zeller T. (2018). Predictive value of long-term changes of growth differentiation factor-15 over a 27-year-period for heart failure and death due to coronary heart disease.. PLoS One.

[62] Xu J., Kimball T.R., Lorenz J.N. (2006). GDF15/MIC-1 functions as a protective and antihypertrophic factor released from the myocardium in association with SMAD protein activation.. Circ. Res..

[63] Heldin C.H., Miyazono K., ten Dijke P. (1997). TGF-β signalling from cell membrane to nucleus through SMAD proteins.. Nature.

[64] Yazawa H., Fukuda T., Kaneda H. (2020). Association of serum growth differentiation factor-15 with eGFR and hemoglobin in healthy older females.. Int. J. Cardiol. Heart Vasc..

[65] Garimella P.S., Katz R., Patel K.V. (2016). Association of serum erythropoietin with cardiovascular events, kidney function decline, and mortality.. Circ. Heart Fail..

[66] Kim D., Lee G.Y., Choi J.O. (2019). Prognostic values of novel biomarkers in patients with AL amyloidosis.. Sci. Rep..

[67] Okada M., Misumi Y., Masuda T. (2021). Plasma growth differentiation factor 15: A novel tool to detect early changes of hereditary transthyretin amyloidosis.. ESC Heart Fail..

[68] Unsicker K., Spittau B., Krieglstein K. (2013). The multiple facets of the TGF-β family cytokine growth/differentiation factor-15/macrophage inhibitory cytokine-1.. Cytokine Growth Factor Rev..

[69] Lim J.H., Jeon Y., Ahn J.S. (2021). GDF-15 predicts in-hospital mortality of critically ill patients with acute kidney injury requiring continuous renal replacement therapy: A multicenter prospective study.. J. Clin. Med..

[70] Nair V., Robinson-Cohen C., Smith M.R. (2017). Growth differentiation factor-15 and risk of CKD progression.. J. Am. Soc. Nephrol..

[71] Perez-Gomez M.V., Pizarro-Sanchez S., Gracia-Iguacel C. (2021). Urinary Growth Differentiation Factor-15 (GDF15) levels as a biomarker of adverse outcomes and biopsy findings in chronic kidney disease.. J. Nephrol..

[72] Jaradat J.H., Nashwan A.J. (2023). Cardiovascular-kidney-metabolic syndrome: Understanding the interconnections and the need for holistic intervention.. J Med Surg Public Health.

[73] Nogueira-Machado J.A., Chaves M.M. (2008). From hyperglycemia to AGE-RAGE interaction on the cell surface: A dangerous metabolic route for diabetic patients.. Expert Opin. Ther. Targets.

[74] Li J., Yang L., Qin W., Zhang G., Yuan J., Wang F. (2013). Adaptive induction of growth differentiation factor 15 attenuates endothelial cell apoptosis in response to high glucose stimulus.. PLoS One.

[75] Higuchi K., Masaki T., Gotoh K. (2007). Apelin, an APJ receptor ligand, regulates body adiposity and favors the messenger ribonucleic acid expression of uncoupling proteins in mice.. Endocrinology.

[76] Nakayasu E.S., Syed F., Tersey S.A. (2020). Comprehensive proteomics analysis of stressed human islets identifies GDF15 as a target for type 1 diabetes intervention.. Cell Metab..

[77] Eizirik D.L., Pasquali L., Cnop M. (2020). Pancreatic β-cells in type 1 and type 2 diabetes mellitus: Different pathways to failure.. Nat. Rev. Endocrinol..

[78] Al-kuraishy H.M., Al-Gareeb A.I., Alexiou A. (2022). Metformin and growth differentiation factor 15 (GDF15) in type 2 diabetes mellitus: A hidden treasure.. J. Diabetes.

[79] Kadoglou N.P.E., Tsanikidis H., Kapelouzou A. (2010). Effects of rosiglitazone and metformin treatment on apelin, visfatin, and ghrelin levels in patients with type 2 diabetes mellitus.. Metabolism.

[80] Eddy A.C., Trask A.J. (2021). Growth differentiation factor-15 and its role in diabetes and cardiovascular disease.. Cytokine Growth Factor Rev..

[81] Jung S.B., Choi M.J., Ryu D. (2018). Reduced oxidative capacity in macrophages results in systemic insulin resistance.. Nat. Commun..

[82] Gerstein H.C., Pare G., Hess S. (2016). Growth differentiation factor 15 as a novel biomarker for metformin.. Diabetes Care.

[83] Mazagova M., Buikema H., van Buiten A. (2013). Genetic deletion of growth differentiation factor 15 augments renal damage in both type 1 and type 2 models of diabetes.. Am. J. Physiol. Renal Physiol..

[84] Johnen H., Lin S., Kuffner T. (2007). Tumor-induced anorexia and weight loss are mediated by the TGF-β superfamily cytokine MIC-1.. Nat. Med..

[85] Suriben R., Chen M., Higbee J. (2020). Antibody-mediated inhibition of GDF15–GFRAL activity reverses cancer cachexia in mice.. Nat. Med..

[86] Breit S.N., Manandhar R., Zhang H.P., Lee-Ng M., Brown D.A., Tsai V.W.W. (2023). GDF15 enhances body weight and adiposity reduction in obese mice by leveraging the leptin pathway.. Cell Metab..

[87] Wang J., Wei L., Yang X., Zhong J. (2019). Roles of growth differentiation factor 15 in atherosclerosis and coronary artery disease.. J. Am. Heart Assoc..

[88] Minamino T., Orimo M., Shimizu I. (2009). A crucial role for adipose tissue p53 in the regulation of insulin resistance.. Nat. Med..

[89] Kim Y., Noren Hooten N., Evans M.K., Stimulates C.R.P. (2018). CRP stimulates GDF15 expression in endothelial cells through p53.. Mediators Inflamm..

[90] Benichou O., Coskun T., Gonciarz M.D. (2023). Discovery, development, and clinical proof of mechanism of LY3463251, a long-acting GDF15 receptor agonist.. Cell Metab..

[91] Xiong Y., Walker K., Min X. (2017). Long-acting MIC-1/GDF15 molecules to treat obesity: Evidence from mice to monkeys.. Sci. Transl. Med..

[92] Fung E., Kang L., Sapashnik D. (2021). Fc-GDF15 glyco-engineering and receptor binding affinity optimization for body weight regulation.. Sci. Rep..

[93] Lim S., Kim D-H., Yang J. (2022). 235-LB: YH34160, a novel long-acting GDF15 fusion protein, exerts potent and sustained body weight loss in rodent obesity models.. Diabetes.

[94] Zhang S.Y., Bruce K., Danaei Z. (2023). Metformin triggers a kidney GDF15-dependent area postrema axis to regulate food intake and body weight.. Cell Metab..

[95] Kempf T., Wollert K.C. (2009). Growth differentiation factor-15: A new biomarker in cardiovascular disease.. Herz.

[96] Lind L., Wallentin L., Kempf T. (2009). Growth-differentiation factor-15 is an independent marker of cardiovascular dysfunction and disease in the elderly: Results from the Prospective Investigation of the Vasculature in Uppsala Seniors (PIVUS) Study.. Eur. Heart J..

[97] Dominguez-Gonzalez C., Badosa C., Madruga-Garrido M. (2020). Growth differentiation factor 15 is a potential biomarker of therapeutic response for TK2 deficient myopathy.. Sci. Rep..

[98] Hagström E., James S.K., Bertilsson M. (2016). Growth differentiation factor-15 level predicts major bleeding and cardiovascular events in patients with acute coronary syndromes: Results from the PLATO study.. Eur. Heart J..

[99] Liu H., Wang H., Tao L. (2013). Stress-induced growth-differentiation factor 15 plays an intriguing role in cardiovascular diseases.. Chin. Med. J. (Engl.).

[100] Wollert K.C., Kempf T. (2012). Growth differentiation factor 15 in heart failure: An update.. Curr. Heart Fail. Rep..

[101] Montoro-García S., Hernández-Romero D., Jover E. (2012). Growth differentiation factor-15, a novel biomarker related with disease severity in patients with hypertrophic cardiomyopathy.. Eur. J. Intern. Med..

[102] Kahli A., Guenancia C., Zeller M. (2014). Growth differentiation factor-15 (GDF-15) levels are associated with cardiac and renal injury in patients undergoing coronary artery bypass grafting with cardiopulmonary bypass.. PLoS One.

[103] Eggers K.M., Kempf T., Lind L. (2012). Relations of growth-differentiation factor-15 to biomarkers reflecting vascular pathologies in a population-based sample of elderly subjects.. Scand. J. Clin. Lab. Invest..

[104] Abdellah M.A., Hassen H.A., Abdel-Hafiz A-R.M., Fouad D.A., Youssef A.A., Idriss N.K. (2020). Growth differentiation factor 15 in patients with acute coronary syndrome and its relation to type 2 diabetes mellitus.. Egypt. J. Hosp. Med..

[105] He X., Su J., Ma X. (2020). The association between serum growth differentiation factor 15 levels and lower extremity atherosclerotic disease is independent of body mass index in type 2 diabetes.. Cardiovasc. Diabetol..

[106] Carlsson A.C., Nowak C., Lind L. (2020). Growth differentiation factor 15 (GDF-15) is a potential biomarker of both diabetic kidney disease and future cardiovascular events in cohorts of individuals with type 2 diabetes: A proteomics approach.. Ups. J. Med. Sci..

[107] Frimodt-Møller M., von Scholten B.J., Reinhard H. (2018). Growth differentiation factor-15 and fibroblast growth factor-23 are associated with mortality in type 2 diabetes – An observational follow-up study.. PLoS One.

[108] Na K.R., Kim Y.H., Chung H.K. (2017). Growth differentiation factor 15 as a predictor of adverse renal outcomes in patients with immunoglobulin A nephropathy.. Intern. Med. J..

[109] Ham Y.R., Song C.H., Bae H.J. (2018). Growth differentiation factor-15 as a predictor of idiopathic membranous nephropathy progression: A retrospective study.. Dis. Markers.

[110] Thorsteinsdottir H., Salvador C.L., Mjøen G. (2020). Growth differentiation factor 15 in children with chronic kidney disease and after renal transplantation.. Dis. Markers.

[111] Wang J., Han L.N., Ai D.S. (2023). Growth differentiation factor 15 predicts cardiovascular events in stable coronary artery disease.. J. Geriatr. Cardiol..

[112] Kopytsya MP, Petyunina OV, Vyshnevska IR, Tytarenko NV, Hilova YV (2016). The role of a new biomarker growth differentiation factor 15 in prognosis of patients with acute coronary syndrome and type 2 diabetes mellitus.. Bull KhNU, Med.

[113] Bao X., Borné Y., Muhammad I.F. (2019). Growth differentiation factor 15 is positively associated with incidence of diabetes mellitus: The malmö diet and cancer–cardiovascular cohort.. Diabetologia.

[114] Shin M.Y., Kim J.M., Kang Y.E. (2016). Association between growth differentiation factor 15 (GDF15) and cardiovascular risk in patients with newly diagnosed type 2 diabetes mellitus.. J. Korean Med. Sci..

[115] Kempf T., Guba-Quint A., Torgerson J. (2012). Growth differentiation factor 15 predicts future insulin resistance and impaired glucose control in obese nondiabetic individuals: Results from the XENDOS trial.. Eur. J. Endocrinol..

